# Executive Functioning as Mediator in the Longitudinal Relationship Between Media Multitasking and Divergent Thinking in Adolescents

**DOI:** 10.1002/pchj.70038

**Published:** 2025-07-12

**Authors:** Shuyu Shan, Ziying Li, Yuxin Fan, Xinru Zhao, Xiuya Lei, Yidi Chen

**Affiliations:** ^1^ School of Humanities and Social Sciences Beijing Forestry University Beijing China; ^2^ Shenzhen Campus Jinan University Guangzhou China

**Keywords:** divergent thinking, executive function, media multitasking, mediating effect, middle school students

## Abstract

Junior high school students frequently multitask with media because of the rapid development of media tools. It is vital to investigate the relationship between junior high school students' individual cognitive abilities and media multitasking to better support their educational and developmental needs. Using a longitudinal design, this study investigated the relationship between media multitasking and divergent thinking, and the mediating role of executive function. Creativity and media were measured using the Development of Adolescent Executive Function Scale, the Alternative Uses Test (AUT), and the Media Multitasking Scale (MMS). Six hundred and nine junior high school students were assessed twice within a six‐month period (at T1 and T2). After controlling for grade, gender, and place of origin, T1 media multitasking was negatively correlated with T2 divergent thinking and T2 executive function. Moreover, T2 executive function was negatively correlated with T2 divergent thinking. Middle schoolers' T1 media multitasking significantly negatively predicted their T2 divergent thinking *β = −*0.1. Vertically, T2 executive function partially mediates the relationship between T1 media multitasking and T2 divergent thinking. High media multitasking reduces individual executive function, whereas low executive function can improve individual divergent thinking. This study reveals the relationship between media multitasking and divergent thinking, as well as the longitudinal mediating mechanism of executive function. Media multitasking can negatively predict divergent thinking, and T2 executive function had a significant longitudinal mediating effect on the relationship between T1 media multitasking and T2 divergent thinking.

## 1 Introduction

The speed of people's lives has accelerated in recent years owing to rapid economic development and technological advancements. Multimedia chores are now the standard for people's everyday work, study, and personal lives. Media multitasking is the simultaneous or rapid alternating of multiple tasks, at least one of which is related to media use (Li et al. 2023). According to a study conducted in the United States, 66% of teenagers “often” or “sometimes” listen to music on their mobile phones or computers while doing their homework; 60% of students “often” or “sometimes” read text messages on their mobile phones while completing homework; and 50% “often” or “sometimes” use social networking sites while doing their homework (Wartella et al. 2016). These findings suggest that media multitasking is prevalent in adolescents' daily lives. Domestic studies have also shown that more than half of adolescents engage in media multitasking in their daily lives (Luo et al. 2018).

The development of technology is inseparable from a country's innovation ability (Shi et al. 2021). As the reserve army of national innovative talents, the creativity of young people is of great significance to a country's development. Dechaume et al. (2024) believe that the core of creativity is creative thinking, including convergent thinking and divergent thinking. Research has shown that media multitasking reduces divergent thinking, which is an important component of creativity (Loh and Lim 2020; Mehta et al. 2012). Divergent thinking refers to an individual using unusual ways to generate multiple answers from a given piece of information (Li et al. 2023). Studies have shown that media multitasking is negatively correlated with individual attention, memory, and divergent thinking (Madore et al. 2020; Loh and Lim 2020); therefore, paying attention to media multitasking can help schools optimize teaching methods, prevent excessive use of media multitasking (Junco and Cotten 2012; Lei 2023; Li et al. 2024), enhance young people's innovative ability, and cultivate innovative talents.

### 1.1 The Relationship Between Media Multitasking and Divergent Thinking

In recent years, many researchers have explored the relationship between media multitasking and divergent thinking through questionnaires and experiments (Kühnel et al. 2017; Madjar and Oldham 2006). Previous studies have examined the relationship between media multitasking and individual divergent thinking (Kapadia and Melwani 2021; Loh and Lim 2020). Much research stated that multimedia tasks negatively predict divergent thinking. Kapadia and Melwani (2021) set up an experimental media multitasking situation to explore the effects of multitasking on individuals' divergent thinking in task processing. Individuals in the media multitasking context produced more original ideas. Liu et al. (2022) showed that the level of media multitasking is negatively correlated with divergent thinking among high media multitaskers. Shane‐Simpson and Bakken's (2024) study on college students also confirmed the negative correlation between the two. According to the creative investment theory, creativity is a kind of investment portfolio composed of multiple factors. Sternberg (1985) proposed that “creativity is the rational investment of people into five internal resources, including personality and motivation, and this investment mode is also affected by the environment.” Environmental factors such as culture, education, and social support influence creativity through individual ability factors. Creativity investment theory also emphasizes the interaction between individual and environmental factors. Media use is increasingly popular, and modern teenagers have more access to media and use it for a longer time. Media has become an essential way for teenagers to learn about the world and socialize (Yin et al. 2023). Additionally, media information is intensive, and teenagers make more use of various fragmented time to frequently switch between different media tasks for learning and socializing (Zamanzadeh and Rice 2021). Media multitasking also increasingly affects the development of individual abilities and cognition; thus, it may be one of the environmental factors affecting divergent thinking. Individuals with high levels of media multitasking will show characteristics that hinder individual creativity (Shi et al. 2024).

However, there are also inconsistent conclusions about the relationship between multimedia tasks and divergent thinking. Ophir et al. (2009) measured and compared the divergent thinking of heavy media and light media multitaskers and found no significant difference between multimedia tasks and divergent thinking. Ophir et al. (2009) divided 33 subjects into two groups: 16 heavy media multitaskers and 17 light media multitaskers; however, the sample sizes were too small, which affected the significance of the results. A study by Loh and Lim (2020) using the median split analysis technique found that the fluency and originality scores of heavy media multitaskers on the Unconventional Uses Test were significantly higher than those of light media multitaskers, providing new evidence for a correlation between media multitasking and divergent thinking. Zhou et al. (2024) found that individual handling with media multitasking can show higher creativity. According to cognitive load theory, individuals will have cognitive processing activities when using social media functions or acquiring information. When these resources need to be processed and the processing exceeds the total amount of cognitive resources possessed by the individual, this will lead to conflicts between this overloaded information and functions and people's limited cognitive resources (Liu et al. 2019). High cognitive load will enhance distractions and lead to the decline of attention (Gorman and Green 2016; Xu et al. 2024), which in turn leads to the decline of cognitive abilities such as creativity. However, it also allows the brain to flexibly reorganize information to produce more creative behaviors through mind wandering (Drody et al. 2022; Zhang et al. 2023).

Creative investment theory indicated that media multitasking declines divergent thinking (Sternberg and Lubart 1996). Due to the lack of supporting empirical evidence, the causal relationship between the two is unclear, as are the internal and intrinsic mechanisms involved. Therefore, this study aims to explore the negative effect of media multitasking on divergent thinking in adolescents. Moreover, a few pieces of evidence indicated that people may be oriented to different levels of creativity after a decline in attention, and the mechanisms of media multitasking decline in divergent thinking remain to be explored.

### 1.2 The Mediating Role of Executive Functions

As media multitasking has become increasingly common, more attention has been paid to its influence on cognition (Alho et al. 2022; Kong et al. 2023; Luo et al. 2022). Numerous studies have explored the effects of media multitasking on executive function, finding important influences on attention, working memory, and inhibitory control (Zhao et al. 2024; Rioja et al. 2023; Murphy and Shin 2022). In terms of attention, Ophir et al. (2009) showed that individuals high in media multitasking experience attention deficits and unresponsiveness, as well as constant eye movement, which produces ocular distraction. In terms of working memory, Minear et al. (2013) found that media multitasking reduces the capacity and processing speed of an individual's working memory. In terms of inhibitory control, one study found that participants who completed both video games and audio search tasks performed worse on inhibitory control (Bailey and Uijtdehaage 2015). Among them, inhibitory control, working memory, and cognitive flexibility are the core components of executive function (Diamond 2013). Previous studies have shown that these cognitive abilities are closely related to media multitasking and divergent thinking. Pollard and Courage (2017) showed that people who frequently multitask media have greater working memory capacity. However, some researchers proposed that this effect may be related to the frequency of media multitasking in an inverted U‐shaped curve, that is, only people who engage in a medium amount of media multitasking have better working memory performance (Shin et al. 2020). A study published in *Nature* showed that media multitasking negatively affects an individual's working memory, particularly through their attentional states (Madore et al. 2020). The multitasking theory proposed by Ophir et al. (2009) holds that frequent multitasking leads to distraction, which affects cognitive ability and working memory. This may reduce the brain's executive function in the long term (Lawson and Mayer 2024). Media multitasking also reduces individuals' inhibitory control and cognitive flexibility (Bailey and Uijtdehaage 2015). Seddon et al. (2021) found that better media multitasking ability is associated with better cognitive flexibility task performance.

However, the relationship between executive function and divergent thinking remains unclear. Some studies have suggested that executive function is positively correlated with divergent thinking (Lin et al. 2021). Beaty et al. (2016) found that individuals with high executive function performed better in divergent thinking. One study explored the relationship between cognitive flexibility in multitasking and creativity through four experiments, in which multitasking improved creativity through the synergistic effect of activation and cognitive flexibility (Kapadia and Melwani 2021). However, other studies found that improvements in executive function have an unstable effect on creative performance. This may have been influenced by situational factors. For example, a study of 21 college students found no significant improvement in performance during a creative task despite training on working memory (Sawyer and Willis 2011). Further, there are also studies showing that low executive function can improve an individual's divergent thinking levels. Shi et al. (2024) showed that individuals with high levels of media multitasking tend to have low inhibitory control ability. Individuals with low inhibitory control have challenges in maintaining attention and switching between different levels of inhibitory control; this may affect the cognitive flexibility of individuals. Moreover, high levels of media multitasking may also reduce individuals' working memory; this, in turn, affects individual creativity. Several studies have shown that media multitasking can reduce individual executive function (Madore et al. 2020; Tao and Tao 2022).

According to the two‐channel theory of creativity, two parallel pathways exist to achieve creativity: flexibility and persistence pathways (Nijstad et al. 2010). The flexibility pathway emphasizes the dispersion of attention. This lower cognitive control state allows more internal and external stimuli to flood the cognitive processing system, enabling the brain to flexibly reorganize information to produce creative behaviors (Zhou et al. 2024). Media multitasking provides the soil for flexible pathways. Frequent task switching weakens cognitive control, producing an influx of a large amount of endogenous information, thus enabling the flexible reorganization of external stimulus information in the brain. Individuals with stronger executive function can better suppress stereotyped thinking and use and switch attention more flexibly (Chen et al. 2024). This enhances individual divergent thinking and produces more creative behavior.

According to previous studies, executive function plays a mediating role between media multitasking and divergent thinking. Among them, media multitasking can reduce individual executive function, and the relationship between executive function and divergent thinking is uncertain. This study also hopes to explore the relationship between individual executive function and divergent thinking through longitudinal research.

### 1.3 The Present Study

**FIGURE 1:**
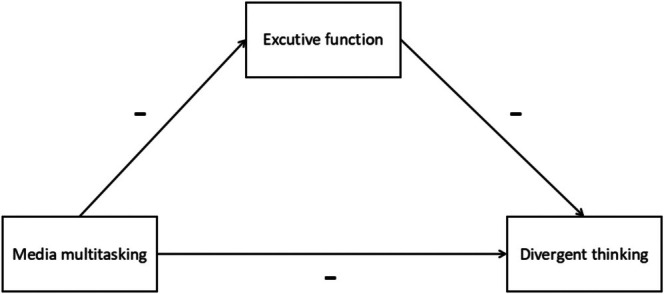
The hypothesis model.

According to the creativity investment theory, the development of creativity is influenced by environmental factors. Previous studies have shown that the situational variable of media multitasking is strongly associated with the three major components of executive functioning (Lu et al. 2017; Seddon et al. 2021). Executive functioning, a factor of individual cognitive abilities, has been shown to have a double‐sided impact on divergent thinking (Davidson et al. 2006; Dreu et al. 2011; Zabelina and Robinson 2010). Moreover, according to the dual‐channel theory of creativity, media multitasking can activate the flexibility pathway of creativity, reduce individual executive function, promote the distraction of attention, and then affect individual divergent thinking levels. Executive function plays a mediating role between media multitasking and divergent thinking. Thus, the relationship among executive function, media multitasking, and divergent thinking is relatively complex. Based on this theoretical assumption and the lack of current research in related fields focusing on the junior high school student population, the present study constructed a mediation model through a longitudinal tracing research design to explore the causal relationship between media multitasking and divergent thinking in junior high school students, along with the internal mechanisms involved. To this end, the following hypothesis is proposed (Figure 1):

Hypothesis 1 *Media multitasking negatively predicts divergent thinking*.

Hypothesis 2 *Executive function at T2 significantly mediates the longitudinal relationship between media multitasking at T1 and divergent thinking at T2; that is, executive function significantly mediates the relationship between media multitasking and divergent thinking*.

## 2 Material and Methods

### 2.1 Participants

The first phase of this study was conducted from the end of December 2021 to January 2022 (T1), adopting the whole cluster sampling method. Thirteen classes were offered at a Jiangxi Province middle school, and 707 paper questionnaires were distributed. Excluding 33 invalid questionnaires due to incomplete or highly repetitive responses, the final number of valid questionnaires was 674, with an effective recovery rate of 95.33%.

Previous studies have explored the changes in executive function of senior primary school children within 6 months (Ai et al. 2024); thus, this study conducted a 6‐month longitudinal study. In June 2022 (T2), the second administration of the test was carried out on 13 classes of students to whom paper questionnaires were distributed. Tests from 98 students were excluded because they did not complete the first test, did not respond to items in the second test, or had too many repetitive answers. This resulted in 609 valid questionnaires, with an effective recovery rate of 86.14%. Table 1 presents the participants' demographic characteristics.

**TABLE 1:** Demographic characteristics of subjects of the study.

	Form	Quorum (T1)	Proportion (%) (T1)	Quorum (T2)	Proportion (%) (T2)
Sex	Male	335	49.7	296	48.6
	Women	339	50.3	313	51.4
Grade	Six	326	48.4	298	48.9
	Seven	348	51.6	311	51.1
Place of origin	Municipalities	89	13.2	77	12.6
	Countryside	585	86.8	532	87.4
Age		14.20 ± 0.99		14.17 ± 0.96	

### 2.2 Procedures

The test was administered to the class as a group in the classroom by a teacher who had been trained in the correct procedures. Before test administration, the primary tester explained the purpose of the study to the class using a standardized PowerPoint presentation. After obtaining students' and their guardians' informed consent, the tester explained in detail how the questionnaire should be filled out, as well as any precautions that needed to be taken. After ensuring the students understood the above, paper questionnaires were distributed. The students were asked to fill in the questionnaires based on their own circumstances. The whole study was approved by the Human Study Ethics Committee of Beijing Forestry University.

All participants completed the following tests in the same order: (1) demographic information (name, grade, class, birth year, gender, and birthplace); (2) the Media Multitasking Scale (MMS); (3) the Development of Adolescent Executive Function Scale; and (4) the Alternative Uses Test (AUT). After administering the tests, the main tester thanked the students for their participation by distributing small snacks and stationery as compensation for their time.

### 2.3 Measurement Tools

1. Media Multitasking Scale (MMS): This scale was developed for Chinese adolescents by Luo et al. (2018) with reference to the media multitasking questionnaires of Ophir et al. (2009) and Baumgartner et al. (2014, 2017). The scale consists of 14 questions divided into three sections measuring adolescents' media/media multitasking (5 items), media/non‐media multitasking (4 items), and preoccupation (5 items). Seven media and four non‐media activities were involved. The scale includes questions such as “I can concentrate on walking and not look at electronic products such as mobile phones.” A five‐point Likert scale ranging from “*never*” to “*always*” was used, with five reverse‐scored questions. Higher total scores indicated that adolescents multitasked more frequently with media. The Cronbach's coefficient for this study was 0.829 for T1 and 0.838 T2.
2. Alternative Uses Test (AUT): Cheng et al. (2022) used the Chinese AUT questionnaire to measure Chinese divergent thinking. The same AUT scale used in this study, which asks participants to report as many novel uses of an everyday object as possible (Runco et al. 2016). Participants were asked to respond to “unconventional, creative, and unusual” uses of paper clips and barrels, listing as many as possible for 3 min per question. The more original and unique, the better. The participants' answers were scored on three dimensions: fluency, adaptability, and originality (Guilford 1967; Silvia et al. 2008). In this study, Cronbach's coefficient was 0.941 for T1, with the questions referring to “paper clip” and “barrel.” It was 0.936 for T2, with questions referring to “brick” and “newspaper.”
3. Development of Adolescent Executive Function Scale (Huang et al. 2014) was used to measure the junior high school students' executive functioning. The scale contains the three dimensions of inhibitory control, cognitive flexibility, and working memory. It consists of 21 questions (six measuring inhibitory control, eight measuring cognitive flexibility, and seven measuring working memory). The scale includes questions like “I can't solve problems creatively.” A three‐point scale (1 = “*no*,” 2 = “*sometimes*,” 3 = “*often*”) was used to reverse score the items scores. Higher scores indicated better executive functioning. The scale had a Cronbach's alpha coefficient of 0.851 at T1 and 0.867 at T2. In summary, all tests had good internal consistency reliability across the two administrations.

### 2.4 Data Analysis

SPSS software (version 24.0) was used to analyze the loss rate using an independent samples *t*‐test and Chi‐square test. The correlations between media multitasking, executive function, and divergent thinking during the T1 and T2 phases were explored using covariance analysis. The negative predictive effect of media multitasking on T2 at T1 was explored using hierarchical regression. AMOS software was also used to examine the longitudinal mediating effect of executive function on the relationship between media multitasking and divergent thinking among middle school students.

## 3 Results

### 3.1 Common Method Bias

After eliminating the invalid data, the data were tested for common method bias. Using the Harman one‐way test for common method bias (Chen et al. 2024), the variable explained by the factor with the largest eigenvalue is 19.46%, which is below the critical value of 40%. Therefore, there was no significant common methodological bias in the study variables.

### 3.2 Dropout Analysis

An independent samples t‐test and a chi‐square test for the main variables in the attrition and non‐attrition samples revealed that gender, age, executive function, and divergent thinking did not differ significantly in the pre‐test (*p >* 0.050; see Table 2). However, participants who dropped out were older [*t*(672) = 2.34, *p =* 0.023] and had higher levels of media multitasking [*t*(672) = 2.17, *p =* 0.034]. Further, the sample was a non‐random attrition sample.

**TABLE 2:** T1 and T2 drop‐out rate analysis.

Variant	Clusters	*N*	*M*	SD	*t*	df	*p*
Age	Drop‐out group	65	14.48	1.05	2.34	672.00	0.02
	Non‐ drop‐out group	609	14.18	0.98			
Executive function	Drop‐out group	65	48.45	7.22	−1.05	672.00	0.30
	Non‐ drop‐out group	609	49.38	6.84			
Divergent thinking	Drop‐out group	65	6.12	4.43	−1.52	672.00	0.13
	Non‐ drop‐out group	609	7.07	4.85			
Media multitasking	Drop‐out group	65	34.62	7.67	2.17	672.00	0.03
	Non‐ drop‐out group	609	32.19	8.66			

### 3.3 Co‐Correlation Analysis of Media Multitasking, Executive Functioning, and Divergent Thinking

The results of the co‐correlation analyses of the total media multitasking, divergent thinking, and executive functioning scores from T1 and T2, with grade, gender, and place of origin as covariates, are shown in Table 3. After controlling for grade, gender, and place of origin, media multitasking at T1 was negatively correlated with divergent thinking and executive function at T2 (*r = −*0.16, *p <* 0.001 and *r = −*0.28, *p <* 0.001, respectively). Executive function at T2 was negatively correlated with divergent thinking at T2 (*r = −*0.01, *p =* 0.029).

**TABLE 3:** Descriptive analysis and correlations of media multitasking, executive function, and creativity (*n* = 609).

Variables	*M*	SD	1	2	3	4	5
1. Media multitasking T_1_	32.15	8.65	—				
2. Divergent thinking T_1_	7.08	4.86	−0.13**	—			
3. Divergent thinking T_2_	8.87	6.15	−0.16***	0.42***	—		
4. Executive function T_1_	49.35	6.85	−0.38***	−0.06*	−0.01*	—	
5. Executive function T_2_	49.78	6.74	−0.28***	−0.03*	−0.01*	0.58***	—

* *p* < 0.05.

** *p* < 0.01.

*** *p* < 0.001.

### 3.4 The Role of Media Multitasking in the Longitudinal Prediction of Divergent Thinking

Hierarchical regression analyses were used to examine the longitudinal predictive effects of T1 media multitasking on divergent thinking at T2. Participants' grade, sex, place of origin, and divergent thinking at T1 were used as the first stratified variables, and media multitasking at T1 was introduced as the second stratified variable in the regression analysis.

Table 4 summarizes the results. After controlling for grade, gender, place of origin, and T1 divergent thinking, T1 media multitasking was able to significantly and negatively predict divergent thinking at T2 (*β = −*0.11, *p =* 0.009), with 23% of the variance explained.

**TABLE 4:** Hierarchical regression analysis of media multitasking T1 and creativity T2 (*n* = 609).

Variant	Divergent thinking T_2_	
	First step	Second step
Grade	−0.17***	−0.15***
Sex	0.06	0.07*
Place of origin	−0.09*	−0.09*
Divergent thinking T_1_	0.41***	0.40***
Media multitasking T_1_		−0.11**
*F*	43.06***	36.60***
*R* ^2^	0.22***	0.23**
*ΔR* ^2^		0.23**

* *p* < 0.05.

** *p* < 0.01.

*** *p* < 0.001.

### 3.5 Delay‐Mediated Effects of Executive Functioning Between Media Multitasking and Divergent Thinking

To explore the longitudinal relationship between media multitasking, executive functioning, and divergent thinking, data from T1 and T2 were integrated and analyzed.

**FIGURE 2:**
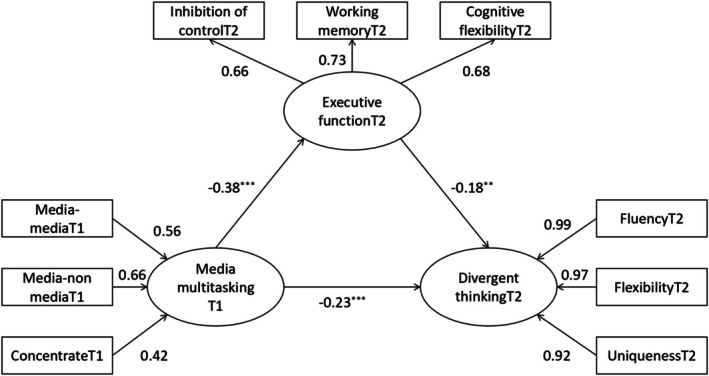
Tests for mediating effects of executive function T2 in the relationship between media multitasking T1 and divergent thinking T2.

To test the longitudinal mediating role of executive function in the relationship between media multitasking and divergent thinking among middle school students, Amos 26.0 was used. The model fit was good (see Table 5). Table 6 and Figure 2 show the results. The direct effect of media multitasking at T1 on T2 divergent thinking was significant (*β = −*0.23, *p <* 0.001). Media multitasking at T1 significantly negatively predicted executive functioning at T2 (*β = −*0.38, *p <* 0.001), and executive functioning at T2 significantly negatively predicted divergent thinking at T2 (*β = −* 0.18, *p =* 0.010). In other words, T2 executive function significantly mediated the longitudinal mediation between the relationship of T1 media multitasking and T2 divergent thinking. This is further evidence that media multitasking not only directly predicts divergent thinking but also works through the indirect path of executive function.

**TABLE 5:** Fitting index of the mediating model between the relationship between T2 of executive function and T1 of media multitasking and T2 of divergent thinking.

Goodness‐of‐fit indicator	*X* ^2^/df	CFI	NFI	IFI	TLI	RMSEA
Fitness index	2.447	0.989	0.982	0.989	0.984	0.049

**TABLE 6:** Tests for mediating effects of executive function T2 in the relationship between media multitasking T1 and divergent thinking T2.

Effect	Standardized effect size	SE	Bootstrapping			
			Bias‐corrected 95% CI		Percentile 95%	
			Lower	Upper	Lower	Upper
Total effect	−0.164	0.056	−0.275	−0.054	−0.276	−0.054
Indirect effect	0.070	0.024	0.034	0.130	0.029	0.122
Direct effect	−0.234	0.062	−0.357	−0.116	−0.356	−0.115

## 4 Discussion

A longitudinal follow‐up design with a time interval of 4 months was used to explore the mediating role of executive function and its subcomponents in the relationship between media multitasking and divergent thinking among Chinese junior high school students. This study aimed to verify whether the mediating model of executive function holds true, and to further clarify and compare in detail the relationships among media multitasking, executive function, and divergent thinking through analyzing longitudinal data. The Media Multitasking Scale was used to reflect individuals' frequency of media multitasking, the Non‐Conventional Uses Test was used to characterize divergent thinking, and the Adolescent Executive Functioning Scale was added to measure junior high school students' daily executive functioning. This study found the following results: (1) when controlling for grade level, gender, and place of origin, media multitasking was significantly and negatively correlated with both executive functioning and divergent thinking, while executive functioning was also negatively correlated with divergent thinking; (2) junior high school students' media multitasking at T1 was able to significantly and negatively predict divergent thinking at T2, which supported Hypothesis 1 and indicated a negative relationship between media multitasking and divergent thinking; and (3) executive function at T2 played a partial mediating role between media multitasking at T1 and divergent thinking at T2.

### 4.1 Media Multitasking Undermines Adolescents' Divergent Thinking

Media multitasking has become the new norm in contemporary middle school students' daily lives (Li and Fan 2022), and its effects on individuals' divergent thinking have received widespread attention (Kapadia and Melwani 2021; Loh and Lim 2020). Media multitasking can negatively predict divergent thinking in adolescents, which is consistent with previous research results (Liu et al. 2022; Loh and Lim 2020; Zhou et al. 2024). According to the principle of splitting attention, if individuals are asked to divide their attention among different features of various learning stimuli, learning will decrease (Cutting et al. 2020). According to the cognitive load theory, when people are exposed to a large amount of information, the brain will be unable to effectively process too much information, resulting in problems such as inattention and reduced decision‐making quality (Xu et al. 2024). Media multitasking will cause individuals to divide their attention among different tasks (Brand et al. 2022; Drody et al. 2023), which will lead to individual cognitive fatigue and decline in executive power, and reduce individual creative insight (Pham and Sanocki 2024). When media multitasking, individuals are exposed to irrelevant information that competes with the information that the individual is trying to focus on, causing the individual to constantly select and allocate attention (Uncapher and Wagner 2018). This distracts their attention, which leads to the reduction of individual attention resources and creative awareness, and cognitive fatigue, thereby resulting in the reduction of individual divergent thinking. In addition, due to the popularity of electronic products, teenagers have a high level of media multitasking (Madore et al. 2020). Previous research results show that media multitasking can significantly negatively predict creativity of heavy media multitasking compared with other groups (Liu et al. 2022; Ophir et al. 2009). Therefore, the negative prediction of media multitasking on divergent thinking in this study may also be related to the fact that teenagers are heavy media multitaskers, and excessive media multitasking leads to a significant decrease in individual creativity. In this study, the correlation between T2's divergent thinking and executive function, and T1's media multitasking and T2's divergent thinking was small. In previous studies, media multitasking was not significantly correlated with the fluency dimension of divergent thinking (Zhou et al. 2024), nor was divergent thinking significantly correlated with general cognition (Chen et al. 2024). The possible reason is that this study focused on junior high school students, whose divergent thinking and executive functions are still in the developing stage. At the same time, the influence of media multitasking and executive function on divergent thinking is a complex double‐edged relationship, which also has an impact on relevance (Xu et al. 2024; Zhou et al. 2024).

### 4.2 Explanatory Mechanisms for Executive Functions

The results show that executive function plays a mediating role in the relationship between media multitasking and divergent thinking. Vertically, media multitasking at T1 significantly and negatively predicted the level of executive function at T2. Further, executive function negatively affected the level of divergent thinking at T2. Some studies have shown that executive function may limit the development of divergent thinking (Chrysikou et al. 2011) because a high level of working memory and inhibitory control will limit the fluency and flexibility of an individual's thinking. Thus, that individual's thinking is limited, and their imagination and creativity are reduced. Media multitasking situations can lead to reduced attention, concentration, inhibitory control, and working memory, which in turn limit that individual's divergent thinking (Chrysikou et al. 2011). Therefore, the levels of individual divergent thinking are reduced, which affects creativity. Frequent task switching in media multitasking weakens individual executive functions and causes a large amount of endogenous information to flood in, thus enhancing the individual mental‐wandering phenomenon (Huang et al. 2024; Matthews et al. 2022).

According to the current attention theory, the defocusing attention feature of mental wandering is likely to contribute to individual creative problem solving. In a wandering state of mind, a large amount of endogenous information emerges, providing an important source of uniqueness and richness in thinking. This enhances individual divergent thinking and creativity (Zhou et al. 2024). Therefore, an increase in media multitasking reduces an individual's executive and cognitive functions; this includes inhibitory control, working memory, and cognitive flexibility, thereby increasing an individual's level of divergent thinking. Media multitasking leads to a decline in inhibitory control (Healey et al. 2013), thus activating the flexibility pathway of creativity, which will further promote the influx of stimuli into the processing system and improve the development of individual creative thinking, especially divergent thinking. In addition, low working memory capacity leads to poor cognitive inhibition and difficulty in continuously devoting attention resources; however, this is consistent with the flexibility pathway's emphasis on achieving jump connections between distant concepts through distraction, thus promoting the development of divergent thinking (Jarosz et al. 2012). Media multitasking leads to the reduction of individual cognitive flexibility, which will reduce attention span and improve the persistence of individual cognition and attention (Cragg and Chevalier 2012; Ie et al. 2012; Li et al. 2023). This helps individuals improve the fluency of divergent thinking. Media multitasking reduces inhibitory control, working memory, and cognitive flexibility, which in turn promotes divergent thinking level development.

However, the direct effect was much larger than the indirect effect. First, media multitasking may have a greater negative effect on individual divergent thinking. Second, executive function may only play a partial mediating role, and other cognitive functions such as cognitive fatigue and attention distraction may play a mediating role (Fisher et al. 2023; Pham and Sanocki 2024), thereby leading to the reduction of divergent thinking levels. Finally, dependency theory suggests that prolonged exposure to a particular form of media may lead to an individual's dependence on that medium, which affects performance in other aspects of life (Sparrow et al. 2011). Adolescents are more likely to use media products, and heavy media multitaskers are more dependent on media, which has a greater negative impact on divergent thinking (Liu et al. 2022; Loh and Lim 2020).

### 4.3 Implications

#### 4.3.1 Theoretical Implications

This study explored the relationship between media multitasking and creativity from the dual perspectives of media psychology and the psychology of creativity. Moreover, it explored the roles executive functions and their subcomponents play in this context, thus expanding the existing body of empirical knowledge at a theoretical level.

This study examined the relationship between middle school students' media multitasking abilities and creativity. It innovatively explored the internal mechanisms of executive functioning, explored the double‐side effect of media multitasking on divergent thinking from a new perspective, and it expanded the relevant theoretical research. Most previous studies used a cross‐sectional design (Duff et al. 2014), whereas this study explored the causal relationship and mechanisms involved in the relationship between media multitasking and creativity through a tracing design. Further, previous related studies mostly investigated college students, with little attention being paid to younger adolescent participants, whose exposure to and use of mobile phones and other electronic devices is more common on a daily basis (Becker et al. 2013; Deng et al. 2022; Ettinger and Cohen 2020). Therefore, this study explored the impact of media multitasking on adolescents' divergent thinking, which will help us better understand and interact with them.

#### 4.3.2 Practical Implications

This study investigated the effects of media multitasking and its underlying mechanisms on creativity in adolescents. These findings can help adolescents, parents, and schools all recognize the effects of media multitasking and adopt appropriate ways to guide adolescents in reducing their use of electronic media.

Although media multitasking can promote the development of individual divergent thinking by reducing executive function, the direct effects were larger than the indirect effects. Therefore, it is still necessary to manage and control adolescents' media multitasking. In the future, schools and families should work together to reduce media multitasking in teenagers' daily lives. Schools can establish classroom norms and regulations and help students develop more hobbies and interests by stimulating their interest in learning and divergent thinking. This will help keep them from becoming addicted to electronic media (Shane‐Simpson and Bakken 2024). Schools should also pay attention to cultivating innovative talents, optimize teaching methods in teaching, and prevent students from excessive media multitasking behavior (Soldatova and Koshevaya 2023; Toyama and Hayashi 2021). Parents can cultivate the correct knowledge and abilities regarding media multitasking when educating junior high school students and cultivating independent divergent thinking and problem solving (Zhou and Deng 2023; Pousada et al. 2023). To this end, they can encourage their children to learn and explore independently and to play freely. When children's executive function is decreasing, teachers and parents can also encourage students to do more creative activities to help stimulate the divergent thinking of teenagers and enhance their creativity. Junior high school students themselves can mitigate the impact of media multitasking by establishing self‐supervision and control mechanisms, such as using tools and apps to help them better manage their time and required tasks or to set a specific time to periodically disconnect from media while they study so they can focus on their work instead of being constantly distracted (Shin et al. 2024).

### 4.4 Limitations and Future Directions

This study has the following limitations. Firstly, in terms of research methodology, both media multitasking and executive functioning relied on students' self‐reports. This may have introduced some bias, and laboratory experiments or more ecological measures could be used in the future.

Second, in terms of research content, the present study is a preliminary exploration of the internal mechanisms involved in the relationship between media multitasking and divergent thinking. However, in real life, executive function may not be the only mediating factor and may be influenced by other factors (Kapadia and Melwani 2021). Thus, it is not feasible to examine the relationship between executive functioning and media multitasking from a single perspective. In the future, attempts should be made to explore whether the interactions between other factors and executive function may have different effects on divergent thinking.

Third, in terms of the research participants, this study only used seventh‐ and eighth‐grade students from three middle schools in Jiangxi Province as survey participants. These students tended to be homogeneous and lacked diversity in terms of intelligence level and study habits, which biased the results to a certain extent. In the future, it will be necessary to ensure the participation of adolescent participants from different geographic regions and different age groups, while maintaining an even distribution of participants by sex, class, and place of birth, to minimize the influence of participant demographic variables on the correlations between the variables.

## Conflicts of Interest

The authors declare no conflicts of interest.
